# Antibiotic therapy in patients with high prostate-specific antigen: Is it worth considering? A systematic review

**DOI:** 10.1080/2090598X.2019.1677296

**Published:** 2019-10-25

**Authors:** Diaa-Eldin Taha, Omar M. Aboumarzouk, Islam Osama Koraiem, Ahmed A. Shokeir

**Affiliations:** a Department of Urology, Faculty of Medicine, Kafrelsheikh University, Kafrelsheikh, Egypt; b Glasgow Urological Research Unit, Department of Urology, Queen Elizabeth University Hospital, Glasgow, UK; c Dentistry and Nursing, School of Medicine, University of Glasgow, Glasgow, UK; d Department of Urology, Damanhour International Medical Institute, Beheira, Egypt; e Department of Urology, Urology and Nephrology Centre, Mansoura University, Mansoura, Egypt

**Keywords:** Antibiotic therapy, prostate-specific antigen (PSA), prostate biopsy (PBx), non-steroidal anti-inflammatory drugs (NSAIDs)

## Abstract

**Objective**: To address the question of whether antibiotic therapy can obviate the need for prostate biopsy (PBx) in patients presenting with high prostate-specific antigen (PSA) levels.

**Methods**: With the increase in unnecessary PBx in men with high PSA levels, a systematic review was performed according to the Cochrane Reviews guidelines and in accordance with the Preferred Reporting Items for Systematic Reviews and Meta-Analyses (PRISMA) checklist.

**Results**: The literature search yielded 42 studies, of which 11 were excluded due to irrelevance of data. Most of the studies were retrospective, nine studies were randomised controlled trials, and there were seven prospective non-randomised trials. The age range of the patients was 51–95 years. Antibiotics, predominantly ofloxacin or ciprofloxacin, combined with a non-steroidal anti-inflammatory drug (NSAID) or not, were prescribed for 2–8 weeks. All studies focussed on PSA levels ranging from ≤ 4 to ≥ 10 ng/mL. Furthermore, antibiotic therapy normalised PSA levels by a wide variety of percentages (16–59%), and the PSA level decrease also varied widely and ranged from 17% to 80%. For patients who had unchanged or decreased PSA, carcinoma was found in 40–52% and 7.7–20.3%, respectively. No cancer was detected if the PSA level decreased to < 4 ng/mL.

**Conclusion**: Antibiotic therapy is clinically beneficial in patients with high PSA levels. PSA reduction or normalisation after medical therapy, either antibiotic and/or NSAID, for ≥ 2 weeks can avoid unnecessary PBx. Antibiotic therapy is more beneficial when the PSA level is < 20 ng/mL.

**Abbreviations**: EPS: expressed prostatic secretion; PBx: prostate biopsy; (%f)(f/t)(t)PSA, (percentage free) (free/total) (total) serum PSA; PSAD: PSA density; RCT: randomised controlled trial; VB3: voided bladder urine 3

## Introduction

In daily practice, some urologists often prescribe antibiotics before prostate biopsy (PBx) to men with a newly increased PSA to decrease inflammation-induced PSA elevation and help to reduce unnecessary PBx. However, others have reported that antibiotic treatment has no significant effect on the PSA level and that a lowered level of PSA after antibiotic treatment does not mean a decreased risk of prostate cancer [1].

PBx is a potentially morbid procedure. Prostatitis is commonly reported on needle biopsies and 65–70% of patients with abnormal PSA levels do not have cancer on prostate needle biopsy. After a 2-year clinical and biochemical follow-up of symptomatic men who had a high PSA level and a normal DRE, and normal repeat PSA level, PBx can be safely avoided [2].

In the present review we aimed to address the controversy of whether antibiotic treatment can exclude inflammation in the differential diagnosis of PSA elevation and thus can avoid unnecessary PBx. We considered patients with LUTS, normal DRE and normal urine analysis, and elevated PSA levels.

## Methods

### Search strategy and study selection

The systematic review was performed according to the Cochrane Reviews guidelines and in accordance with the Preferred Reporting Items for Systematic Reviews and Meta-Analyses (PRISMA) checklist [3].

The search strategy was conducted to find relevant studies from the Medical Literature Analysis and Retrieval System Online (MEDLINE; 1966–2018), Excerpta Medica dataBASE (EMBASE; 1980–2018), Google Scholar, and individual urological journals. The search was conducted in January 2018.

Terms used included: ‘prostate’, ‘biopsy’, ‘high PSA’, and ‘antibiotic therapy’.

Mesh phrases included:

(‘high PSA’[Mesh]) AND ‘unnecessary prostate biopsy’[Mesh])
((‘high PSA’[Mesh]) AND ‘antibiotic therapy’[Mesh]) AND ‘unnecessary prostate biopsy’[Mesh])
(((‘high PSA’[Mesh]) AND ‘antibiotic therapy’[Mesh]) AND ‘ unnecessary prostate biopsy’[Mesh]) AND ‘NSAID’[Mesh])

All language papers were considered if reporting on PSA reduction after antibiotic therapy. References of searched papers were evaluated for potential inclusion. Authors of the included studies were contacted whenever the data were not available or not clear.

### Inclusion criteria

1. All studies reporting on antibiotic therapy in patients with high PSA levels.
2. Studies published in the English language over the period 1980–2018.

### Exclusion criteria

1. Animal studies and case reports.
2. Studies on patients with high PSA levels without documented antibiotic therapy.

Two reviewers (D.T. and O.M.A.) identified all studies that adhered to the inclusion criteria for full review. Each reviewer independently selected studies for inclusion. Disagreement between the extracting authors was resolved by consensus or referred to a third author (A.A.S.).

### Data extraction and analysis

The objectives were to evaluate the efficacy and safety of using antibiotic therapy in PSA reduction resulting in the avoidance of unnecessary PBx. The variables extracted from each study were: patient demographics, antibiotic type, antibiotic duration, NSAID use with antibiotic or not, PSA reduction level after antibiotic therapy, and rate of PBx after antibiotic therapy.

## Results

**Figure 1.**
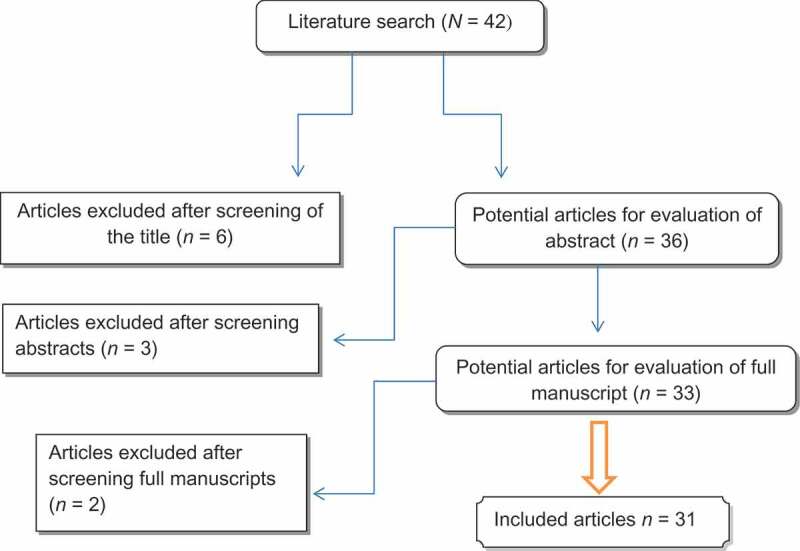
Flowchart of article selection.

The literature search yielded 42 studies, of which 11 were excluded due to irrelevance of data (Figure 1). The majority of studies were retrospective and nine studies were randomised controlled trials (RCTs) [4–12]. Furthermore, there were seven prospective non-randomised trials [13–19]. All studies reported on the variables indicated in the data extraction section and are listed in Table 1 [4–32, 34, 35].

**Table 1.** The detailed features of the studies included in the review.

	Authors	Study type	Journal and publication year	No. of patients	PSA level, ng/mL (unless otherwise stated)	Inflammation type	Antibiotic duration	Antibiotic type	NSAID	Cancer detection rate after antibiotic, %
1	Busato et al. [14]	Prospective non-randomised	Int Braz J Urol. 2015	106	4–10	Asymptomatic	3 weeks	Ciprofloxacin 500 mg twice a day	No	25.0
2	Toktas et al. [12]	RCT	J Endourol. 2013	140	2.5–10		3 weeks	Levofloxacin 500 mg	No	16
3	Saribacak et al. [8]	RCT	Int J Clin Exp Med. 2014	100	4–10	Acute	4 weeks	Ofloxacin 400 mg	No	8.3
4	Kyung et al. [30]	Retrospective	Int Neurourol J. 2010	107	4–10	Acute	8 weeks	Quinolone antibiotic	No	NA
5	Lee et al. [7]	RCT	Korean J Urol. 2012	413	4–10	Chronic	8 weeks	Quinolone antibiotic	No	11.6
6	Azab et al. [4]	RCT	Transl Androl Urol. 2012	142	> 4	Chronic	6 weeks	Ofloxacin 400 mg/day	Piroxicam 20 mg/day	21.8
7	Erol et al. [5]	RCT	Urol Int. 2006	97	> 4 ng/dL	Acute	2–3 weeks	Ciprofloxacin 500 mg twice daily	Diclofenac sodium 75 mg slow-release once a day	NA
8	Ozden et al. [33]	Retrospective	Int Urol Nephrol. 2007	52	Inflammation: Grade 1: 2.4 Grade 2: 5.2 Grade 3: 5.7	Asymptomatic	Assess degree of inflammation after TURP	No antibiotics	No	NA
9	Bozeman et al. [25]	Retrospective	J Urol. 2002	95	> 4	Chronic	4 weeks	Fluoroquinolones, trimethoprim-sulfamethoxazole or doxycycline	Ibuprofen was the most often prescribed. Celecoxib was prescribed when patients had any history of intolerance to NSAIDs or peptic ulcer disease	25.5
10	Bulbul et al. [19]	Prospective	J Med Liban. 2002	48	5.0–28.5	Asymptomatic	2 weeks	Ciprofloxacin	No	NA
11	Kaygisiz et al. [27]	Retrospective	Prostate Cancer Prostatic Dis. 2006	48	4–10	Asymptomatic	3 weeks	Yes	No	10.8
12	Nadler et al. [31]	Retrospective	J Urol. 1995	148	> 4	Asymptomatic	No	No antibiotics	No	NA
13	Morote et al. [26]	Retrospective	Eur Urol. 2000	284	Group 1: 7.8 Group 2: 6.7 Group 3: 6.4	BPH vs chronic vs acute	No	No antibiotics	No	NA
14	Irani et al. [35]	Retrospective	J Urol. 1997	66		BPH tissue	Assess degree of inflammation	No antibiotics	No	NA
15	Schaeffer et al. [9]	RCT, double blinded	J Urol. 2005	377	8.33 ± 4.46	Chronic	28 days	Levofloxacin vs ciprofloxacin	No	NA
16	Karazanashvili et al. [15]	Prospective	Eur Urol. 2001	61	4–10	Acute	15 days	Mainly ofloxacin (400 mg, 2 times/day,	No	NA
17	Lorente et al. [16]	Prospective	Int J Biol Markers. 2002	90	4–20	Acute	3 weeks	Ofloxacin	No	NA
18	Baltaci et al. [13]	Prospective	J Urol 2009	100	4–10	Asymptomatic	20 days	Ofloxacin, 400 mg	No	29.4
19	Serretta et al. [17]	Prospective	Prostate Cancer Prostatic Dis. 2008	99	4–10		3 weeks	Ciprofloxacin, 500 mg	No	20.3
20	Heldwein et al. [6]	RCT	BJU Int. 2011	245	> 2.5	Asymptomatic	30 days	Levofloxacin 500 mg	No	15.8
21	Kobayashi et al. [24]	Retrospective	Urol Int. 2008	51	> 4	Asymptomatic	4 weeks	Levofloxacin 300 mg/day	No	NA
22	Del Rosso et al. [23]	Retrospective	Urologia. 2012	31	4–10	Asymptomatic	2 weeks	Ciprofloxacin 1000 mg	Ketoprofen 100 mg rectally	28.5
23	Faydaci et al. [22]	Retrospective	Actas Urol Esp. 2012	108	> 2.5	Acute	3 weeks	NA	No	NA
24	Kim et al. [18]	Prospective, observational study	Korean J Urol. 2011	86	> 4	Chronic prostatitis	4 weeks	Ciprofloxacin, 500 mg/day	Zaltoprofen 80 mg, three times a day	13.3–26.5 according to PSA level
25	Shtricker et al. [10]	RCT	Int Braz J Urol. 2009	135 (65 received antibiotic)	4–10	Acute	10–14 days	Ofloxacin or ciprofloxacin	No	12
26	Magri et al. [29]	Retrospective	Arch Ital Urol Androl. 2007	471	> 4	Cat. II, III or IV chronic bacterial prostatitis	6 weeks	Combined 500 mg/day ciprofloxacin, 500 mg/day azithromycin	No	29
27	Stopiglia et al. [11]	RCT	J Urol. 2010	98	2.5–10	Category IV prostatitis	4 weeks	Ciprofloxacin 500 mg twice a day (49 patients)		26.9
28	Dirim et al. [28]	Retrospective	Urol Int. 2009	85	> 2.5	Acute	4 weeks in 63 patients 6 weeks in 16 patients 8 weeks in 6 patients	71 received levofloxacin 500 mg once daily and 14 received ciprofloxacin 500 mg twice daily	No	7.7–16.7 according to PSA or f/tPSA
29	Huang et al. [21]	Retrospective	Zhonghua Nan Ke Xue. 2012	150	4–50	Type IIIA histological prostatitis	4 weeks	Ciprofloxacin + Ningbitai and Yunnan Baiyao capsule	No	NA
30	Wang et al. [20]	Retrospective	Zhonghua Nan Ke Xue. 2006	228	> 4	Type IIIA prostatitis	4 weeks	NA	Yes	NA
31	Yoo et al. [32]	Retrospective	Urology. 2014	237	> 2.5	Asymptomatic	Duration not stated	NA (124 patients prescribed antibiotic)	No	2

### Characteristics of the included studies

The 31 included studies were published between 1995 and 2018, and included 4682 patients with an age range between 51 and 95 years.

### The type and duration of antibiotic use

Concerning the duration of antibiotic use, some studies prescribed antibiotics for 2–4 weeks [8–28], whilst others prescribed for 6–8 weeks [4,7,28–30].

Six studies used ofloxacin [4,8,10,13,15,16], six studies used 500 mg ciprofloxacin [5,9,17,19,21,23], and five used levofloxacin [6,9,12,28]. Six studies combined the antibiotic therapy with NSAIDs [4,5,18,20,23,25]. Huang et al. [21] added the plant extracts, Ningbitai and Yunnan Baiyao capsule, to the antibiotic therapy. Magri et al. [29] combined 500 mg/day ciprofloxacin and 500 mg/day azithromycin.

### Effect of antibiotic use

There was no significant difference in the mean change in PSA level between the levofloxacin and ciprofloxacin groups [9].

Shtricker et al. [10] compared 135 patients who received antibiotics (65) with those who did not (70). The PSA levels decreased by 60% in both groups and at PBx prostate cancer was found in 25% of patients in both groups. In both groups, 40% of the patients had no decrease in PSA levels; however, prostate cancer was found in only two patients (12%) who received antibiotics, and in eight (42%) who did not receive antibiotic. PSA levels tend to fall when measurement is repeated after 45 days, regardless of antibiotic use [6].

In the Lee et al. [7] study of 413 patients, 215 (52%) patients had positive findings on expressed prostatic secretion (EPS) or voided bladder urine 3 (VB3) tests. After 8 weeks of quinolone antibiotic therapy, 53 of these 215 men avoided PBx due to of normalisation of their PSA levels.

### No effect of antibiotic on PSA level

Inflammation had no significant influence on total serum PSA (tPSA) level or the percentage free PSA (%fPSA) [26]. The tPSA, %fPSA, and free/total PSA ratio (f/tPSA) alterations before and after antibiotic therapy did not show any statistically significant difference (*P* > 0.05) [22]. There is no advantage in administering antibacterial therapy with initial PSA levels of 4–10 ng/mL, without overt evidence of inflammation [10].

### The studied level of PSA

All studies focussed on PSA levels ranging from 4 to 10 ng/mL. Some studies assessed PSA levels <4 ng/mL [6,18] and others assessed levels >10 ng/mL [9,16,19,21].

The majority of studies addressed the effect of antibiotics on the acutely inflamed prostate, whilst some of the other studies reported on documented chronic inflamed type [4,7,9,18,20,21,25,29].

Morote et al. [26] assessed benign tissue without inflammation in association with chronic prostatitis or acute prostatitis, whilst other studies focussed on patients presenting with LUTs only with normal urine analysis [6,13,19,23,24,27,31,32].

Three studies did not use antibiotics but assessed the degree of inflammation after prostatectomy [33–35].

### The degree of PSA decline

The PSA level was normalised by a wide variety of percentages and varied across the studies from 16% to 59% [7,8,20,25,29,30]. The PSA level decrease also varied widely and ranged from 17% to 80% [4,13,15,18,20,23,24,29,32].

In the Dirim et al. [28] study, PSA levels decreased after antibiotic treatment in 47 of 85 patients. The f/tPSA ratio decreased or remained unchanged in 21 of these 47 cases and increased in 26. There were 38 patients who had increased PSA levels after antibiotic therapy. The f/tPSA ratios decreased or remained unchanged in 20 of these 38 cases and increased in 18. In the Toktas et al. [12] study, there were significant changes in the values of PSA and its derivatives in the antibiotic treatment group, from 5.31 to 4.69 and 4.58 ng/mL, consecutively. In the Kyung et al. [30] study, the PSA density (PSAD) after antibiotic treatment was normalised (< 0.15 ng/mL/mL) in 23 of the 40 patients with a high PSAD before treatment.

Significantly, the mean (range) PSA level decreased by 33.8% from 8.12 (4.02–24.8) to 5.37 (1.35–12.94) ng/mL after treatment (*P *= 0.001) [18], and by 36.4% from 8.48 ng/mL before to 5.39 ng/mL after treatment (*P* < 0.001) [25]. Similarly, in the Wang et al. [20] study, the mean PSA decreased from 6.24 ng/mL before treatment to 4.58 ng/mL 4 weeks after treatment (*P* < 0.05). In the Toktas et al. [12] study, there were significant changes in the values of PSA and its derivatives in the treatment group (from 5.31 to 4.69 and 4.58 ng/mL, consecutively).

### Rate of carcinoma and pbx avoidance

As regard patients who had unchanged or decreased PSA levels, carcinoma was found in 40–52% and 7.7–20.3%, respectively. No cancer was detected if the PSA level decreased to < 4 ng/mL or by > 70% [10,17,27,28]. However, the possibility of prostate cancer in patients with a PSA level of < 2.5 ng/mL is still present [18].

Pathological studies of PBxs after antibiotic therapy revealed prostate cancer in 20.9–25.5%, chronic inflammation in 50.7–74.4%, and BPH in 4.7–21.8% [4,18,25].

With regard to PSA levels, Azab et al. [4] reported that of their 142 patients treated with antibiotic and NSAIDs for 6 weeks, prostate cancer was detected in 12% (three of 25 patients) with PSA levels of < 2.5 ng/mL, 12.7% (six of 47 patients) with PSA levels of ≥ 2.5–< 4.0 ng/mL, and in 30% (21/70 patients) with PSA levels ≥ 4.0 ng/mL.

Shtricker et al. [10] studied the cancer detection rate in patients with PSA levels of 4–10 ng/mL, who received antibiotic therapy (65 patients) vs those who did not (70 patients). The cancer detection rate at PBx in patients who did not have a PSA level decrease was 12% (two of 17 patients) after antibiotic therapy vs 42% (eight of 19 patients) in those no antibiotic therapy [10]. Similarly, Kaygisiz et al. [27] reported that prostate cancer was found at PBx in 10.8% of the patients with PSA levels between 4 and 10 ng/mL, but in none with PSA levels <4 ng/mL.

In the Yoo et al. [32] study, PBx was performed in 50 of 237 patients (21.1%), and only a single case (2%) of prostate cancer was diagnosed. In the Baltaci et al. [13] study, in 17% of the men the tPSA after treatment was < 4 ng/mL and of these five (29.4%) had prostate cancer at PBx.

In the Lee et al. [7] study, the total prostate cancer detection rate was 20.7% in the patients with negative findings on EPS or VB3 tests and 3.3% in the patients with positive findings.

## Discussion

Although there is controversy surrounding the value of antibiotics in reducing higher PSA levels, some urologists in daily practice often prescribe antibiotics before PBx to men with a newly increased PSA level. PSA level reduction after antibiotics might identify those patients in whom PBx can be avoided.

Some researchers have found that antibiotic treatment can decrease inflammation-induced PSA elevation and help to reduce unnecessary PBx. Conversely, others have reported that antibiotic treatment has no significant effect on the PSA level, and a lowered PSA level after antibiotic treatment does not mean a decreased risk of prostate cancer [1].

The antibiotic can be prescribed for 2–4 weeks [8–28] or 6–8 weeks [4,7,28–30]. The type of antibiotic used is based on local sensitivities and quinolones are the most frequently used type.

The evidence for inflammation should be addressed before trying antibiotic therapy in patients with high PSA levels. The proof of inflammation can be delineated via EPS [7], symptoms of acute or chronic prostatitis [4,7,9,18,20,21,25,29], and detection of the degree of inflammation after prostatectomy [33,34].

The PSA level in focus for antibiotic therapy ranges from 4 to 10 ng/mL. Some studies assessed PSA levels <4 ng/mL [6,18], whilst others assessed levels >10 ng/mL [9,16,19,21]. In patients with PSA levels higher than the threshold value, definitive treatment should be not postponed for preliminary antibiotic therapy.

After use of antibiotic therapy, the PSA level was normalised by a wide variety of percentages, ranging from 16% to 59% [6–8,10,20,25,29,30]. Furthermore, the range of the PSA level decrease was 17–80% [4,13,15,18,20,23,29,32], or a > 20% decrease from baseline [24].

The f/tPSA ratio rather than tPSA appears to be more helpful in suggesting prostate cancer in cases receiving antibiotic therapy for high PSA levels [12,28,30].

PBx should be considered without trying antibiotic therapy in patients with high PSA values, if a suspicion of prostatitis does not exist [22].

The rate of cancer detection after receiving antibiotic therapy varied from 2% to 29% [4,6,7,10–13,17,18,25,27–29,32].

Carcinoma was found in 40–52% of patients who did not have a PSA decrease. Conversely, a detection rate of 7.7–20.3% was found in patients who had a PSA decrease in comparison with the pre-treatment values [10,17,27,28].

In the context of pathological results after antibiotic therapy, prostate cancer was evident in only 20.9–25.5%, whilst chronic inflammation and BPH was found in 50.7–74.4% and 4.7–21.8%, respectively [4,18,25].

For specific PSA values, prostate cancer was identified in 12% (three of 25 patients) with PSA levels of < 2.5 ng/mL, 12.7% (six of 47 patients) with PSA levels of ≥ 2.5–< 4.0 ng/mL, and in 30% (21/70 patients) with PSA levels ≥ 4.0 ng/mL [4]. While, the cancer detection rate in patients having a PSA level between 4–10 ng/mL was 10.8–12% [10,27].

## Conclusion

Antibiotic therapy is clinically beneficial in patients with high PSA levels. PSA reduction or normalisation after medical therapy, either antibiotic and/or NSAID, for ≥2 weeks can avoid unnecessary PBx. Antibiotic therapy is more beneficial when the PSA level is <20 ng/mL, especially when the evidence for inflammation is not overt.
